# REDD1-dependent GSK3β dephosphorylation promotes NF-κB activation and macrophage infiltration in the retina of diabetic mice

**DOI:** 10.1016/j.jbc.2023.104991

**Published:** 2023-06-29

**Authors:** Siddharth Sunilkumar, Ashley M. VanCleave, Christopher M. McCurry, Allyson L. Toro, Shaunaci A. Stevens, Scot R. Kimball, Michael D. Dennis

**Affiliations:** 1 Department of Cellular and Molecular Physiology, Penn State College of Medicine, Hershey, Pennsylvania, USA; 2 Department of Ophthalmology, Penn State College of Medicine, Hershey, Pennsylvania, USA

**Keywords:** diabetic retinopathy, inflammation, DDIT4, RTP801

## Abstract

Increasing evidence supports a role for inflammation in the early development and progression of retinal complications caused by diabetes. We recently demonstrated that the stress response protein regulated in development and DNA damage response 1 (REDD1) promotes diabetes-induced retinal inflammation by sustaining canonical activation of nuclear transcription factor, NF-κB. The studies here were designed to identify signaling events whereby REDD1 promotes NF-κB activation in the retina of diabetic mice. We observed increased REDD1 expression in the retina of mice after 16 weeks of streptozotocin (STZ)-induced diabetes and found that REDD1 was essential for diabetes to suppress inhibitory phosphorylation of glycogen synthase kinase 3β (GSK3β) at S9. In human retinal MIO-M1 Müller cell cultures, REDD1 deletion prevented dephosphorylation of GSK3β and increased NF-κB activation in response to hyperglycemic conditions. Expression of a constitutively active GSK3β variant restored NF-κB activation in cells deficient for REDD1. In cells exposed to hyperglycemic conditions, GSK3β knockdown inhibited NF-κB activation and proinflammatory cytokine expression by preventing inhibitor of κB kinase complex autophosphorylation and inhibitor of κB degradation. In both the retina of STZ-diabetic mice and in Müller cells exposed to hyperglycemic conditions, GSK3 inhibition reduced NF-κB activity and prevented an increase in proinflammatory cytokine expression. In contrast with STZ-diabetic mice receiving a vehicle control, macrophage infiltration was not observed in the retina of STZ-diabetic mice treated with GSK3 inhibitor. Collectively, the findings support a model wherein diabetes enhances REDD1-dependent activation of GSK3β to promote canonical NF-κB signaling and the development of retinal inflammation.

Diabetic retinopathy (DR) is a significant ocular complication caused by diabetes that can progress to blindness. Recent estimates suggest that by 2045, DR will affect 160.50 million individuals worldwide, resulting in 44.82 million cases of vision-threatening disease (1). The pathogenesis of DR is complicated and multifactorial; however, it is well accepted that inflammation plays a key role in the progression of the retinal pathology that is caused by diabetes (2, 3). Inflammation is a nonspecific response to injury or stress involving a variety of functional and molecular mediators and is generally viewed as a protective immune response. Conversely, chronically sustained proinflammatory conditions, as is seen with diabetes, contribute to disease progression. Indeed, clinical and preclinical studies support that early intervention to suppress inflammatory mediators prevents vision deficits and retinal pathology caused by diabetes (4, 5, 6, 7).

The transcription factor NF-κB has been well studied as a mediator of inflammation in response to diabetes (8, 9). Increased retinal NF-κB activation is observed in both diabetic patients (8) and in preclinical models of type 1 and type 2 diabetes (10, 11, 12, 13). The NF-κB family of transcription factors (RelA [p65], RelB, c-Rel, p50, and p52) form homodimers/heterodimers that control the expression of an array of proinflammatory molecules, including C–C motif chemokine ligand 2 (CCL2), CCL5, interleukin 1β (IL-1β), and the NLRP3 inflammasome (reviewed in Ref. (14)). Inactive NF-κB dimers are sequestered in the cytoplasm by the regulatory protein inhibitor of κB (IκB). Canonical NF-κB signaling depends on activation of the IκB kinase (IKK) complex, which includes two kinase subunits (IKKα/β) and the regulatory subunit NEMO. IKK phosphorylates IκB to promote its proteasomal degradation, thus permitting the nuclear translocation of NF-κB. Activation of the IKK complex and enhanced NF-κB activity in the retina is well established in preclinical rodent models of diabetes (12, 15, 16, 17). However, the early signaling events whereby diabetes contributes to classical activation of NF-κB signaling in the retina remain to be thoroughly defined.

We recently demonstrated that the stress response protein regulated in development and DNA damage response 1 (REDD1, also known as DDIT4/RTP801) is necessary for diabetes-induced retinal inflammation and activation of canonical NF-κB signaling (13). REDD1 is a 25 kDa protein encoded by the DNA damage–inducible transcript 4 (*Ddit4*) gene. REDD1 expression is low in most adult human and mouse tissues but is robustly upregulated in response to a variety of cell stresses (reviewed in Ref. (18)). REDD1 expression in the retina is dominant in Müller glia, where the protein contributes to a failed adaptive response of the retina that includes gliosis, neurodegeneration, and deficits in visual function (19, 20). A number of recent studies establish a role for REDD1 in the development of inflammation and NF-κB activation (21, 22, 23, 24). Notably, REDD1 promotes atypical NF-κB activation in macrophages exposed to lipopolysaccharide through the direct binding and sequestration of IκBα (22). However, such a mechanism of action does not fully account for the REDD1-dependent increase in canonical NF-κB in the retina of diabetic mice. In particular, REDD1 deletion prevented diabetes-induced IKK activation and promoted IκBα expression in the retina of diabetic mice (13). This suggests an alternative mechanism of action whereby REDD1 enhances IKK signaling.

REDD1 acts, at least in part, by recruiting protein phosphatase 2A to facilitate site-specific dephosphorylation of Akt and thus reduce Akt kinase activity (25). Akt directly phosphorylates a number of protein substrates, including glycogen synthase (GS) kinase 3 (GSK3β). GSK3β is constitutively active and principally regulated by the obstruction of substrate recognition that occurs with Akt-dependent phosphorylation (26). Thus, diabetes-induced REDD1 expression promotes signaling through GSK3β. We recently demonstrated a role for REDD1-dependent GSK3 signaling in regulating nuclear localization of the antioxidant transcription factor Nrf2 (27, 28). Herein, we explored a role for REDD1-dependent GSK3β activation in promoting canonical NF-κB signaling and consequently the development of diabetes-induced retinal inflammation.

## Results

### REDD1 promotes retinal immune cell activation and enhanced GSK3β activity in the retina of diabetic mice

**Figure 1:**
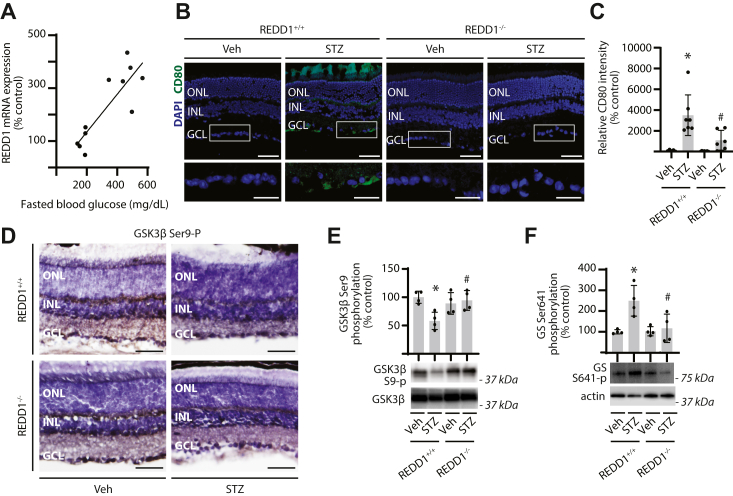
**REDD1 was required forimmune cell activationand GSK3β dephosphorylation in the retina of diabetic mice.** Diabetes was induced in REDD1^+/+^ and REDD1^−/−^ mice by administration of streptozotocin (STZ). All analyses were performed 16 weeks after mice were administered STZ or vehicle (Veh). *A*, fasting blood glucose concentrations were measured. REDD1 mRNA abundance in retinal tissue homogenate was determined by RT–PCR. Correlation between fasting blood glucose and REDD1 mRNA abundance is shown for REDD1^+/+^ mice (Pearson *r* = 0.85). *B* and *C*, CD80-positive cells (*green*) were identified in retinal sections by immunofluorescence. Nuclei were visualized with DAPI (*blue*). Representative micrographs are shown (scale bar represents 50 μm). *White box* indicates area shown below each image at increased magnification (zoom inset scale bar represents 25 μm). Separate images for each channel are provided in Fig. S1. *D*, phosphorylation of GSK3β at S9 was determined by immunolabeling of retinal sections. Representative micrographs are shown (scale bar represents 50 μm). *E*, GSK3β phosphorylation at S9 was evaluated in retinal lysates by Western blotting. Representative blots are shown. Molecular mass in kilodalton is indicated at *right* of each blot. *F*, glycogen synthase (GS) phosphorylation at S641 was evaluated in retinal lysates by Western blotting. Individual data points are plotted with values presented as means ± SD (n = 3–6). Differences between groups were identified by two-way ANOVA. ∗*p* < 0.05 *versus* Veh; #*p* < 0.05 *versus* REDD1^+/+^. DAPI, 4′,6-diamidino-2-phenylindole; GCL, ganglion cell layer; GSK3β, glycogen synthase kinase 3β; INL, inner nuclear layer; ONL, outer nuclear layer; REDD1, regulated in development and DNA damage response 1.

Prior studies support increased REDD1 expression in the retina of diabetic mice (13, 20, 27). Indeed, after 16 weeks of streptozotocin (STZ)-induced diabetes, fasted blood glucose concentrations were increased in coordination with retinal REDD1 mRNA abundance (Fig. 1*A*). Immune cell infiltration was assessed by evaluating CD80-positive cells in retinal sections by immunofluorescence. Diabetic REDD1^+/+^ mice exhibited increased CD80-positive cells in the inner retina; however, an increase in CD80-positive cells was not observed in the retina of diabetic REDD1^−/−^ mice (Figs. 1, *B* and *C* and S1). In diabetic REDD1^+/+^ mice, retinal GSK3β phosphorylation at S9 was attenuated as compared with nondiabetic mice (Fig. 1, *D* and *E*). However, a similar suppressive effect of diabetes on GSK3β was not observed in REDD1^−/−^ mice. Activity of GSK3β was assessed *via* phosphorylation of its downstream target GS at S641. In the retina of diabetic REDD1^+/+^ mice, GS phosphorylation was enhanced as compared with nondiabetic mice (Fig. 1*F*). GS phosphorylation in the retina of diabetic and nondiabetic REDD1^−/−^ mice was similar to that observed in the retina of nondiabetic REDD1^+/+^ mice. The data support REDD1-dependent GSK3β activation in association with immune cell infiltration in the retina of diabetic mice.

### NF-κB activation in Müller glia exposed to hyperglycemic conditions requires REDD1

**Figure 2:**
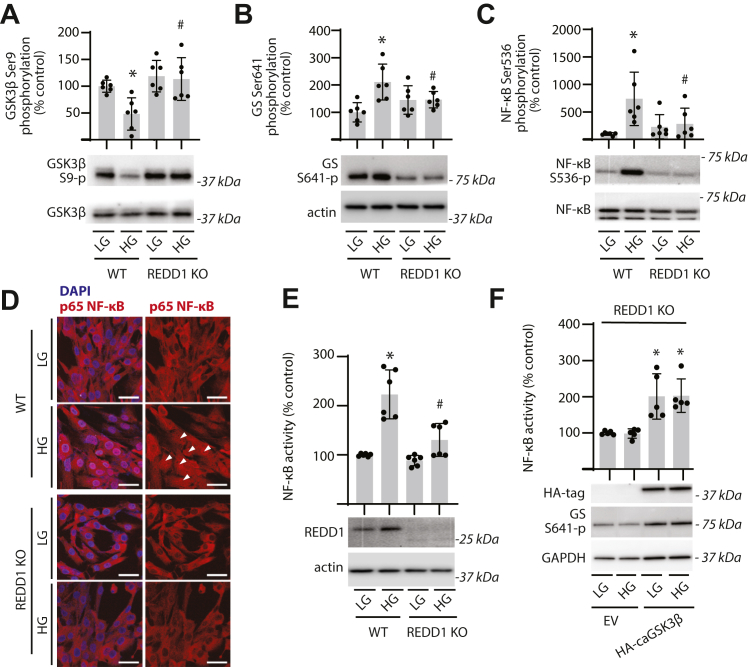
**REDD1 deletion reduced GSK3β dephosphorylation and activation of NF-κBin Müller glial cultures exposed to hyperglycemic conditions.** WT and REDD1 KO human MIO-M1 cells were cultured in medium containing 5.6 mM glucose and exposed to medium containing either 30 mM high glucose (HG) or a low glucose (LG) osmotic control containing 5.6 mM glucose plus 24.4 mM mannitol for 24 h. *A*, GSK3β phosphorylation at S9 was evaluated in cell lysates by Western blotting. Representative blots are shown. Molecular mass in kilodalton is indicated at *right* of each blot. *B*, glycogen synthase (GS) phosphorylation at S641 was evaluated in retinal lysates by Western blotting. *C*, NF-κB phosphorylation at S536 was determined in cell lysates by Western blotting. *D*, nuclear localization of NF-κB p65 (*white arrowheads*) was determined by immunofluorescence. Nuclei were visualized with DAPI (scale bar represents 50 μm). *E*, NF-κB activity was measured in lysates from cells expressing NF-κB firefly luciferase/*Renilla* luciferase reporter plasmids by dual luciferase assay. REDD1 expression was evaluated by Western blotting. *F*, NF-κB reporter activity was evaluated in REDD1 KO cells expressing either an empty vector (EV) control or hemagglutinin (HA)-tagged constitutively active GSK3β S9A (caGSK3β). HA-GSK3β expression and GS phosphorylation at S641 were evaluated by Western blotting. Individual data points are plotted with values presented as means ± SD (n = 5–6). Differences between groups were identified by two-way ANOVA. ∗*p* < 0.05 *versus* LG or EV; #*p* < 0.05 *versus* WT. DAPI, 4′,6-diamidino-2-phenylindole; GSK3β, glycogen synthase kinase 3β; REDD1, regulated in development and DNA damage response 1.

We recently demonstrated that REDD1 expression in the retina is dominant in Müller glia (20). To explore GSK3β signaling in human Müller glia, WT and REDD1 KO MIO-M1 cell cultures were exposed to hyperglycemic conditions for 24 h. In WT cells exposed to hyperglycemic conditions, GSK3β phosphorylation at S9 was attenuated (Figs. 2*A* and S2) and GS phosphorylation at S641 was enhanced (Figs. 2*B* and S2). REDD1 expression was required for the change in phosphorylation of both GSK3β and GS in response to hyperglycemic conditions. In association with the activation of GSK3β, NF-κB phosphorylation at S536 was enhanced in cells exposed to hyperglycemic conditions, and REDD1 was necessary for the effect (Figs. 2*C* and S2). By contrast, REDD1 deletion had no impact on p100 processing or phosphorylation of MAPK p-44/42 at T202/Y204 (Fig. S2). In cells exposed to hyperglycemic conditions, REDD1 was required for increased NF-κB nuclear localization (Fig. 2*D*) and enhanced NF-κB activity (Fig. 2*E*). To evaluate a role for GSK3β in REDD1-dependent NF-κB activation, constitutively active GSK3β S9A variant (caGSK3β) was expressed in REDD1-deficient cells. Expression of caGSK3β enhanced NF-κB activity in REDD1-deficient cells (Fig. 2*F*). Whereas REDD1 deletion prevented increased NF-κB activity in response to hyperglycemic conditions, caGSK3β was sufficient to induce NF-κB activity in the absence of hyperglycemic conditions or REDD1, and there was no additive increase upon exposure to hyperglycemic conditions.

### GSK3β is required for NF-κB activation in response to hyperglycemic conditions

**Figure 3:**
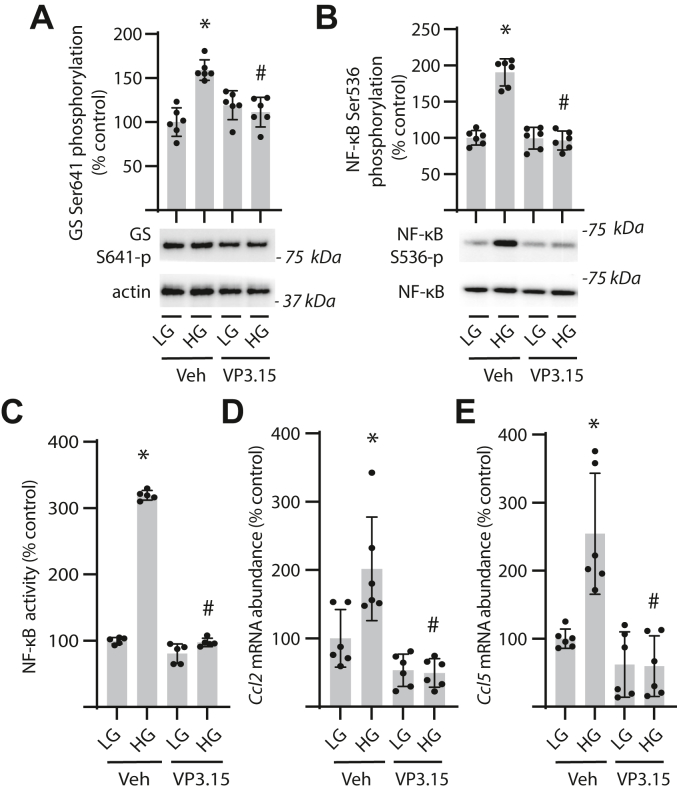
**GSK3 inhibition prevents NF-κBactivation in Müller glial cultures exposed to hyperglycemic conditions.** MIO-M1 cells were cultured in medium containing 5.6 mM glucose and exposed to medium containing either 30 mM high glucose (HG) or a low glucose (LG) osmotic control containing 5.6 mM glucose and 24.4 mM mannitol for 24 h in the presence of either the GSK3 inhibitor VP3.15 or a vehicle (Veh) control. *A*, GS phosphorylation at S641 was quantified in cell lysates by Western blotting. Representative blots are shown. Molecular mass in kilodalton is indicated at *right* of each blot. *B*, NF-κB phosphorylation at S536 was determined in cell lysates by Western blotting. *C*, NF-κB activity was measured in lysates from cells expressing NF-κB firefly luciferase/*Renilla* luciferase reporter plasmids by dual luciferase assay. *D* and *E*, abundance of mRNAs encoding *Ccl2* (*D*) and *Ccl5* (*E*) was determined in cell lysates by RT–PCR. Individual data points are plotted with values presented as means ± SD (n = 6). Differences between groups were identified by two-way ANOVA. ∗*p* < 0.05 *versus* LG; #*p* < 0.05 *versus* Veh. GS, glycogen synthase; GSK3β, glycogen synthase kinase 3β.

**Figure 4:**
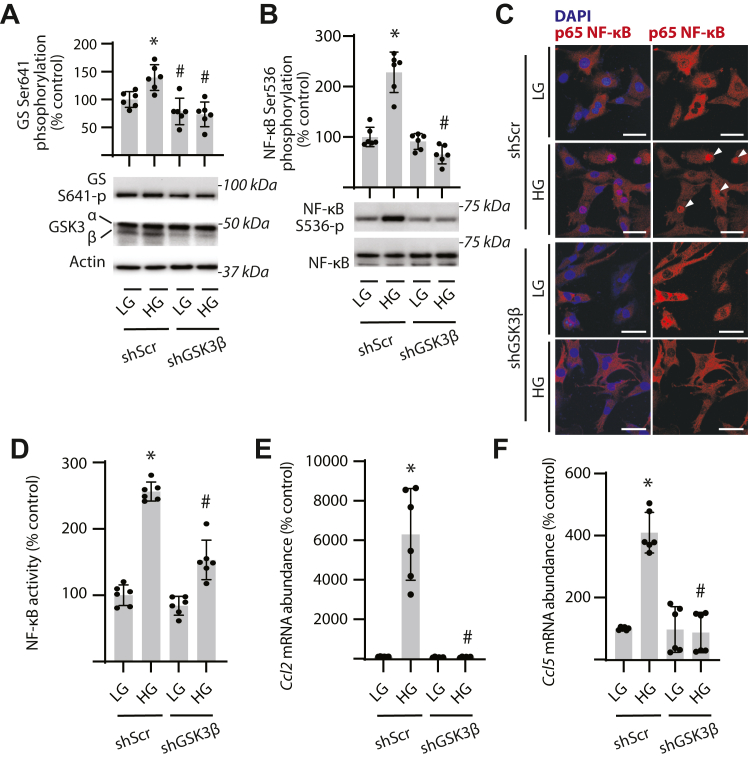
**GSK3β promotes NF-κBactivation and inflammatory cytokine expression in response to hyperglycemic conditions.** GSK3β was knocked down in human MIO-M1 cells by stable expression of an shRNA (shGSK3β). Control cells expressed a scramble shRNA (shScr). Cells were cultured in medium containing 5.6 mM glucose and exposed to medium containing either 30 mM high glucose (HG) or a low glucose (LG) osmotic control containing 5.6 mM glucose plus 24.4 mM mannitol for 24 h. *A*, GS phosphorylation at S641 was assessed in cell lysates by Western blotting. GSK3β expression and phosphorylation at S9 were evaluated by Western blotting. Representative blots are shown. Molecular mass in kilodalton is indicated at *right* of each blot. *B*, NF-κB phosphorylation at S536 was determined in cell lysates by Western blotting. *C*, nuclear localization of NF-κB p65 (*white arrowheads*) was determined by immunofluorescence. Nuclei were visualized with DAPI (scale bar represents 50 μm). *D*, NF-κB activity was measured in lysates from cells expressing NF-κB firefly luciferase/*Renilla* luciferase reporter plasmids by dual luciferase assay. *E* and *F*, abundance of mRNAs encoding *Ccl2* (*E*) and *Ccl5* (*F*) was determined in cell lysates by RT–PCR. Individual data points are plotted with values presented as means ± SD (n = 6). Differences between groups were identified by two-way ANOVA. ∗*p* < 0.05 *versus* LG; #*p* < 0.05 *versus* shScr. DAPI, DAPI, 4′,6-diamidino-2-phenylindole; GS, glycogen synthase; GSK3β, glycogen synthase kinase 3β.

To further investigate a role for GSK3β in NF-κB activation, GSK3 activity was inhibited in Müller cells using VP3.15. GSK3 inhibition prevented enhanced phosphorylation of GS in cells exposed to hyperglycemic conditions (Fig. 3*A*). VP3.15 also prevented an increase in both NF-κB phosphorylation (Fig. 3*B*) and NF-κB activity (Fig. 3*C*) in cells exposed to hyperglycemic conditions. Expression of mRNAs encoding the NF-κB target genes *Ccl2* (Fig. 3*D*) and *Ccl5* (Fig. 3*E*) was reduced in cells exposed to hyperglycemic conditions in the presence of VP3.15. Suppression of NF-κB phosphorylation and IκB degradation in Müller cells exposed to hyperglycemic conditions was also observed with the GSK3 inhibitor CHIR99021 (Fig. S3*A*). As a complementary genetic approach, human Müller glial cell lines with stable GSK3β knockdown (shGSK3β) were generated (Fig. S3, *B* and *C*). In cells expressing an shRNA control, exposure to hyperglycemic conditions increased GS phosphorylation (Fig. 4*A*). GSK3β knockdown reduced GSK3β expression and attenuated GS phosphorylation in both the presence and absence of hyperglycemic conditions. GSK3β knockdown also prevented an increase in NF-κB phosphorylation (Fig. 4*B*) and NF-κB nuclear localization (Fig. 4*C*) in cells exposed to hyperglycemic conditions. Hyperglycemic conditions enhanced NF-κB activity in cells expressing a control shRNA, and GSK3β knockdown suppressed the effect (Fig. 4*D*). Knockdown of GSK3β also prevented an increase in expression of *Ccl2* (Fig. 4*E*) and *Ccl5* (Fig. 4*F*) in response to hyperglycemic conditions. Together, the data support a role for GSK3β in NF-κB activation in Müller glial cells exposed to hyperglycemic conditions.

### GSK3β promotes IKK activation and IκBα degradation

**Figure 5:**
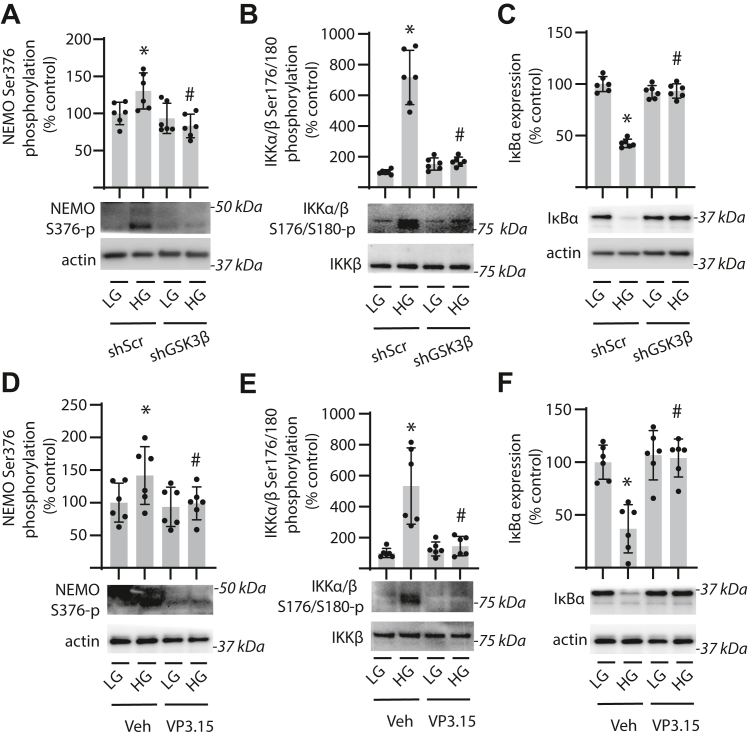
**GSK3β promotes IKK activation in Müller glial cultures exposed to hyperglycemic conditions.***A*–*C*, GSK3β was knocked down in human MIO-M1 cells by stable expression of an shRNA (shGSK3β). Control cells expressed a scramble shRNA (shScr). *D*–*F*, MIO-M1 cells were exposed to culture medium supplemented with VP3.15 to inhibit GSK3 activity or a vehicle (Veh) control. Cells were cultured in medium containing 5.6 mM glucose and exposed to medium containing either 30 mM high glucose (HG) or a low glucose (LG) osmotic control containing 5.6 mM glucose plus 24.4 mM mannitol for 24 h. Phosphorylation of NEMO at S376 (*A* and *D*), phosphorylation of IKKα/β at S176/180 (*B* and *E*), and IκB⍺ expression (*C* and *F*) were determined in cell lysates by Western blotting. Representative blots are shown. Molecular mass in kilodalton is indicated at *right* of each blot. Individual data points are plotted with values presented as means ± SD (n = 6). Differences between groups were identified by two-way ANOVA. ∗*p* < 0.05 *versus* LG; #*p* < 0.05 *versus* shScr or Veh. GSK3β, glycogen synthase kinase 3β; IKK, IκB kinase.

We previously demonstrated that REDD1 sustains canonical NF-κB signaling in Müller glial cells by promoting activation of the IKK complex (13). Hyperglycemic conditions enhanced phosphorylation of NEMO at S376 (Fig. 5*A*) and IKKα/β at S176/S180 (Fig. 5*B*), and GSK3β knockdown prevented the effect. Suppression of GSK3β expression also prevented a reduction in IκBα expression in cells exposed to hyperglycemic conditions (Fig. 5*C*). GSK3β inhibition resulted in a similar suppressive effect on IKK autophosphorylation in cells exposed to hyperglycemic conditions (Fig. 5, *D* and *E*). Reduced IκBα expression in cells exposed to hyperglycemic conditions was also prevented by GSK inhibition (Fig. 5*F*). The data support that GSK3β acts to promote IKK activation and the subsequent degradation of IκBα in response to hyperglycemic conditions.

### GSK3β inhibition prevents NF-κB activation and inflammation in the retina of diabetic mice

**Figure 6:**
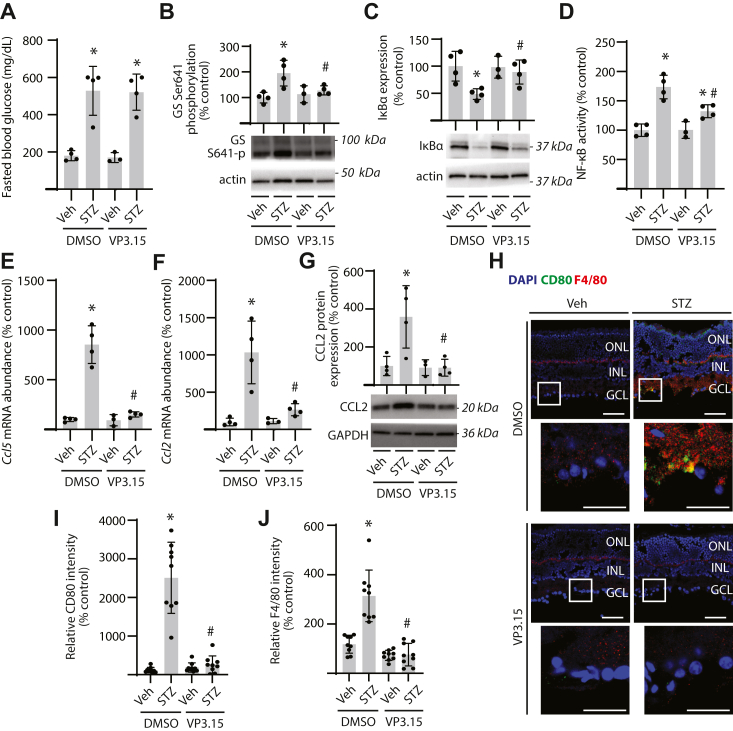
**GSK3 suppression prevents diabetes-induced retinal inflammatory cytokine expression and macrophage infiltration**. Diabetes was induced in mice by administration of streptozotocin (STZ). All analyses were performed 16 weeks after mice were administered STZ or a vehicle (Veh). During the last 3 weeks of diabetes, mice were treated daily by intraperitoneal administration of the GSK3 inhibitor VP3.15 or a Veh (10% DMSO). *A*, fasting blood glucose concentrations were evaluated before tissue collection. *B*, GS phosphorylation at S641 was evaluated in retinal lysates by Western blotting. Representative blots are shown. Molecular mass in kilodalton is indicated at *right* of each blot. *C*, IκB⍺ expression was evaluated in retinal lysates by Western blotting. *D*, NF-κB activity in retinal homogenates was measured by DNA-binding ELISA. *E* and *F*, abundance of mRNAs encoding CCL5 (*E*) and CCL2 (*F*) was determined in retinal lysates by RT–PCR. *G*, CCl2 protein expression was evaluated in retinal lysates by Western blotting. *H*–*J*, CD80 (*green*) and F4/80 (*red*) expression was localized in retinal sections by immunofluorescence. DAPI (*blue*) was used to visualize nuclei. Representative micrographs are shown (scale bar represents 50 μm). *White box* indicates area shown below each image at increased magnification (zoom inset scale bar represents 25 μm). Images depicting separated channels are provided in Fig. S4*B*. Relative expression of CD80 and F4/80 in retinal sections was quantified in *I* and *J*, respectively. Individual data points are plotted with values presented as means ± SD (n = 3–6). Differences between groups were identified by two-way ANOVA. ∗*p* < 0.05 *versus* Veh; #*p* < 0.05 *versus* DMSO. DAPI, 4′,6-diamidino-2-phenylindole; DMSO, dimethyl sulfoxide; GCL, ganglion cell layer; GS, glycogen synthase; GSK3β, glycogen synthase kinase 3β; IκB, inhibitor of κb; INL, inner nuclear layer; ONL, outer nuclear layer.

To investigate a role for GSK3β in diabetes-induced retinal NF-κB activation, 13 weeks after STZ administration, diabetic mice were treated with VP3.15 daily for 3 weeks (Fig. S4*A*). Blood glucose concentrations were elevated by diabetes and not altered by administration of VP3.15 (Fig. 6*A*). Efficacy of GSK3 suppression by VP3.15 was supported by the absence of an increase in GS phosphorylation in retinal tissue lysates of diabetic mice receiving VP3.15 compared with diabetic mice receiving vehicle alone (Fig. 6*B*). GSK3 inhibition attenuated the reduction in IκBα expression (Fig. 6*C*) and suppressed NF-κB activity (Fig. 6*D*) in the retina of diabetic mice. Expression of *Ccl5* (Fig. 6*E*) and *Ccl2* (Fig. 6, *F* and *G*) mRNA was increased in the retina of diabetic mice, and GSK3 inhibition prevented the effect. Diabetes promoted macrophage infiltration into the inner retina, as evidenced by increased CD80 colocalization with F4/80 in retinal sections (Figs. 6*H* and S4*B*). Inhibition of GSK3 suppressed infiltration of both CD80- (Fig. 6*I*) and F4/80-positive (Fig. 6*J*) cells in the retina of diabetic mice. Overall, the data support a role for GSK3β in diabetes-induced retinal inflammation.

## Discussion

**Figure 7:**
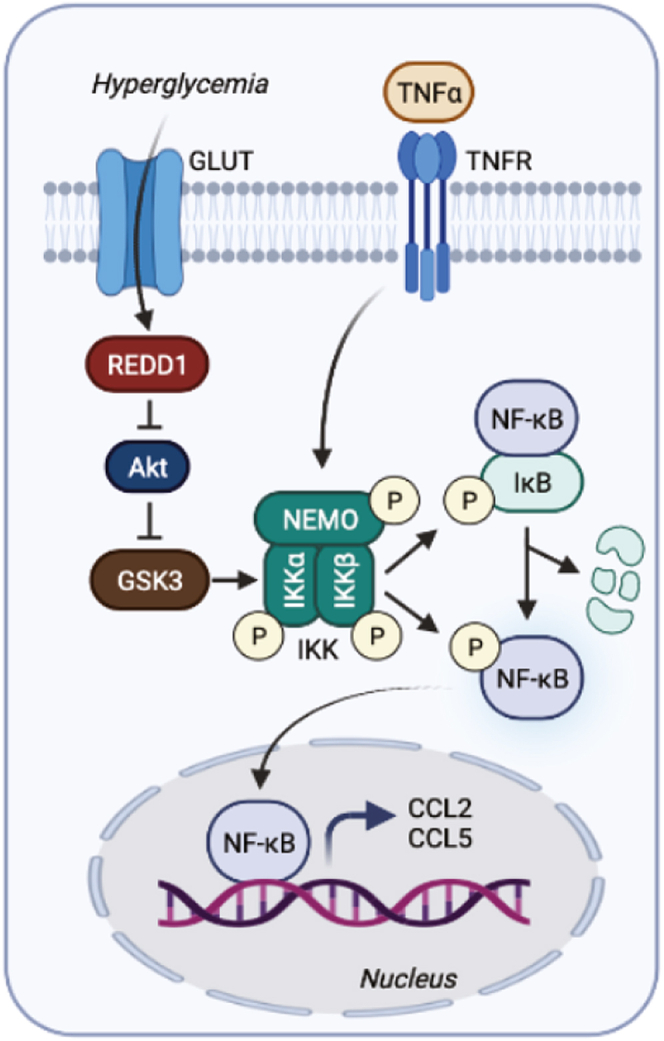
**REDD1-dependent GSK3 signaling contributes to diabetes-induced retinal inflammation.** Working model illustrates role for REDD1 in promoting diabetes-induced GSK3β activation and enhanced NF-κB proinflammatory signaling. Graphic is created with BioRender.com. GSK3, glycogen synthase kinase 3; REDD1, regulated in development and DNA damage response 1.

Studies here identify a role for REDD1-dependent GSK3β activation in regulation of NF-κB signaling and the inflammatory response of the retina to diabetes. We recently demonstrated that REDD1 contributed to diabetes-induced retinal inflammation by promoting canonical NF-κB signaling (13). Herein, increased REDD1 expression was necessary for dephosphorylation of GSK3β in both the retina of STZ-diabetic mice and in Müller glial cultures exposed to hyperglycemic conditions. Diabetes promoted NF-κB activation, enhanced proinflammatory cytokine expression, and led to retinal immune cell activation in a manner that required GSK3β activity. Overall, the findings support a model whereby diabetes-induced REDD1 expression acts to promote retinal inflammation by GSK3β-dependent activation of IKK and thus increased canonical NF-κB signaling (Fig. 7).

Recent evidence supports that pathological changes including gliosis, neuroinflammation, neurodegeneration, and loss of neurovascular coupling precede the clinically visible microvascular abnormalities that define DR (29, 30, 31). DR phenotypes are modeled in mice by 4 to 5 weeks of STZ-diabetes (19, 32, 33) and can be maintained up to 22 months (34). Characteristic pathologies including increased gliosis and neural apoptosis are seen shortly after onset of hyperglycemia (32, 33, 34), with retinal inflammation (13, 35), formation of acellular capillaries, and pericyte ghosts (34) observed after more prolonged disease progression. In the retina, REDD1 is specifically expressed in the Müller glia, where the protein contributes to a failed adaptive response to diabetes resulting in development of functional deficits in vision (20). Müller glia extend radially across the entire retina to provide homeostatic support for all other elements of the retina, including photoreceptors, neurons, and vasculature. REDD1-dependent dephosphorylation of GSK3β was observed throughout the retinal layers in response to diabetes, which is consistent with localization to Müller cells. In response to diabetes, Müller cells become activated and secrete a range of proinflammatory cytokines and adhesion molecules that are target genes of NF-κB (36). We recently demonstrated that REDD1 was required for enhanced NF-κB activity and increased expression of CCL5 and CCL2 in the retina of STZ-diabetic mice (13). Studies here extend on the prior report by demonstrating that diabetes-induced NF-κB activation and enhanced expression of CCL5 and CCL2 in the retina also require GSK3 activity.

The GSK3 kinase family consists of GSK3α and GSK3β paralogs that are encoded by separate genes. While the catalytic domains of GSK3α and GSK3β are nearly identical, their N- and C-terminal regions differ. Identification of a single nucleotide substitution in the ATP-binding pocket recently allowed the development of paralog-specific inhibitors (37). Notably, GSK3β contains a unique N-terminal nuclear localization sequence that allows it to enter the nucleus (38). GSK3β plays a crucial role in the inflammatory response by promoting the expression of proinflammatory cytokines (*e.g.*, tumor necrosis factor alpha [TNFα], IL-1β, CCL2, and CCL5) and downregulating anti-inflammatory cytokine production (*e.g.*, IL-10) (reviewed in Refs. (39, 40)). Inhibition of GSK3β has been pursued as a therapeutic option in preclinical disease models. In a murine model of ischemic injury, administration of the GSK3 inhibitor SB216763 prevented inflammatory symptoms (41). Inhibition of GSK3 activity has also shown therapeutic benefits in protecting against hyperglycemia-induced cardiac inflammation and remodeling in diabetic mice (42). In the present study, VP3.15 prevented an increase in proinflammatory cytokine expression and macrophage infiltration in the retina of diabetic mice. The selection of VP3.15 was based in part on the prior demonstration of its ability to cross the blood retina barrier (43). Moreover, we previously demonstrated that diabetic mice treated with VP3.15 exhibited enhanced activity of the antioxidant transcription factor Nrf2 in retinal lysates, and diabetes-induced oxidative stress in the retina was absent (27). An important caveat to these observations is that at higher concentrations VP3.15 also acts to inhibit phosphodiesterase 7 (44). Notably, VP3.15 and GSK3β knockdown had comparable suppressive effects on proinflammatory cytokine expression in Müller cell cultures exposed to hyperglycemic conditions, suggesting that at the concentration used in these studies it was acting by selectively inhibiting GSK3β.

A growing body of evidence supports a key role for REDD1 in regulating inflammation (13, 21, 22, 23, 24). REDD1 is best known as a dominant regulator of mammalian target of rapamycin 1 (mTORC1) (45). REDD1 inhibits mTORC1 by suppressing GTPase-activating protein activity of the tuberous sclerosis (TSC) complex toward Ras homolog enriched in brain (Rheb) (25, 46). Direct binding of Rheb-GTP, but not Rheb-GDP, to mTORC1 results in its activation (47). However, REDD1 deletion prevents endotoxemia-induced inflammation independently of mTORC1 activation (21). REDD1 acts to suppress TSC complex activity and thus mTORC1 signaling, by targeting protein phosphatase 2A to dephosphorylate Akt on T308 (25). This results in impaired Akt activity toward a number of substrates, including both the TSC complex and GSK3β. In the retina of diabetic mice and in retinal Müller cells exposed to hyperglycemic conditions, REDD1 was required for dephosphorylation of GSK3β, and GSK3 activity was necessary for increased expression of the proinflammatory chemokines CCL2 and CCL5. CCL2 and CCL5 act to recruit leukocytes to the site of inflammation. Indeed, both REDD1 and GSK3 activity were required for macrophage infiltration of the inner retina in diabetic mice.

Lee *et al.* (23) recently provided evidence that REDD1 promotes IKK-independent atypical NF-κB activation in adipocytes of obese rodents by directly binding IκBα to promote nuclear translocation of NF-κB. However, the REDD1-dependent increase in NF-κB activity in the retina of diabetic mice occurs concomitant with attenuated expression of IκBα and enhanced autophosphorylation of IKK (13). Indeed, reduced IκBα expression and enhanced IKK phosphorylation in the retina of diabetic rodents support prior reports (12, 15, 48). While the observation does not exclude atypical NF-κB activation in the context of DR, it suggests that REDD1-dependent NF-κB activity in retina involves conventional NF-κB activation. NF-κB signaling consists of both canonical and noncanonical pathways (reviewed in Refs. (14, 49)). Canonical NF-κB signaling involves activation of IKK complex, which phosphorylates IκBα to stimulate its proteasomal degradation and thus allow nuclear localization of the RelA/p50 heterodimer of NF-κB (50). IKK also directly phosphorylates RelA to promote transactivation (51). In Müller cells exposed to the proinflammatory cytokine TNFα, IKK activity was necessary for REDD1-dependent NF-κB activation (13). In the present study, REDD1 expression was required for dephosphorylation of GSK3β in Müller cells exposed to hyperglycemic conditions, and GSK3 activity was necessary for enhanced IKK autophosphorylation, reduced expression of IκBα, and increased NF-κB activity. Moreover, attenuated expression of IκBα in the retina of diabetic mice was prevented by GSK3 suppression. We did not observe a change in the NF-κB noncanonical pathway, as there was no difference in p100 processing in Müller cell cultures exposed to hyperglycemic conditions. Together with the prior work (13), the data support that increased REDD1 expression and the consequent activation of GSK3β contribute to enhanced NF-κB signaling in the retina of diabetic mice.

NF-κB is a dynamic transcription factor that coordinates complex biological processes involved in the inflammatory response to diabetes (36, 37, 38). A role for GSK3β in regulating the nuclear activity of NF-κB is consistent with several prior reports (52, 53, 54, 55, 56, 57, 58). GSK3β-deficient mouse embryonic fibroblasts exhibit defective NF-κB-mediated gene transcription in response to TNFα (52). Herein, GSK3β was not only required for increased NF-κB activity in Müller cells exposed to hyperglycemic conditions, but expression of a constitutively active GSK3β variant was sufficient to enhance NF-κB activation independently of hyperglycemic conditions or REDD1. In both the retina of diabetic mice and in Müller cells exposed to hyperglycemic conditions, IκBα expression was reduced, and GSK3 inhibition prevented the effect. In Müller cells exposed to hyperglycemic conditions, GSK3β knockdown prevented enhanced IKK autophosphorylation and RelA phosphorylation at S536. This supports that enhanced GSK3β activity promotes NF-κB signaling in response to hyperglycemic conditions by acting upstream of IKK activation and the consequent degradation of IκBα.

While TNFα-induced NF-κB activation consistently depends on GSK3β, evidence supports that the mechanism of action may be independent of IKK (52, 53, 55). For example, the classic GSK3 inhibitor lithium chloride suppresses NF-κB activity without preventing IκBα degradation or the nuclear localization of RelA (53, 55). Evidence supports that GSK3β directly phosphorylates both RelA (53) and IKK (57). Reports have also implicated GSK3 in noncanonical NF-κB signaling through the phosphorylation of p100 (56). Moreover, GSK3β-dependent phosphorylation of the immune signaling adaptor BCL10 (B cell lymphoma/leukemia 10) promotes CARMA1–BCL10–MALT1 complex formation, which is a key signaling event upon antigen receptor engagement of B cells and T cells, and consequently activation of canonical and noncanonical NF-κB pathways (58). Ko *et al*. (59) have also demonstrated a role for GSK3β ubiquitination by TNF receptor–associated factor-6 in regulation of TLR3-mediated proinflammatory cytokine production. TNF receptor–associated factor-6–mediated ubiquitination of GSK3β is essential for TLR3-induced TRIF-assembled multiprotein signaling complex that leads to increased cytokine production *via* NF-κB activation (59, 60). Thus, REDD1-dependent activation of GSK3β is likely to promote NF-κB signaling in the retina of diabetic mice through multiple mechanisms. Conversely, evidence supports that lithium chloride may also promote NF-κB activity in RAW264.7 macrophage cells (61). While silencing of heterogeneous nuclear ribonucleoprotein K, a putative GSK3β interacting protein, abolished lithium chloride–induced NF-κB activation in macrophages, knockdown of heterogeneous nuclear ribonucleoprotein K was not sufficient to prevent NF-κB activation in response to receptor activator of nuclear factor kappa-B ligand; suggesting a GSK3-independent mechanism for activation of NF-κB signaling with lithium chloride administration.

Overall, the findings here provide new insight into the molecular mechanisms that contribute to retinal inflammation with diabetes. These proof-of-concept studies support that diabetes-induced REDD1 expression promotes dephosphorylation of GSK3β and consequently retinal inflammation. At present, there is an unmet need for therapeutics that are preventative and/or provide interventions early in the preclinical and nonproliferative stages of DR by targeting the initiating molecular events that cause retinal pathology. Indeed, therapeutic administration of an siRNA for REDD1 mRNA knockdown has demonstrated promise for improving best-corrected visual acuity in patients with diabetic macular edema (62). However, we recently discovered that the initial increase in retinal REDD1 protein content occurs *via* a post-transcriptional mechanism (63). The observation is significant because it suggests that REDD1 mRNA knockdown is likely to only be partially effective for reducing REDD1 protein expression in the context of DR. In light of the modest benefits seen in diabetic patients treated with an siRNA for retinal REDD1 knockdown, effective suppression of the downstream signaling events whereby REDD1 causes retinal pathology (*i.e.*, GSK3β inhibition) potentially offers an alternative to achieve improved patient outcomes.

## Experimental procedures

### Animals

Male WT (REDD1^+/+^) and REDD1 KO (REDD1^−/−^) B6;129 mice (64) were maintained on a 12:12-h reverse light–dark cycle. Diabetes was induced at 6 weeks of age by administering 50 mg/kg STZ or sodium citrate buffer intraperitoneally for 5 consecutive days. Two weeks postinjection, diabetic phenotype was confirmed by fasting blood glucose concentration >250 mg/dl. To examine a role for GSK3β, C57BL/6J mice (Jackson Laboratory) were administered STZ as described previously and then received daily intraperitoneal injections of either VP3.15 (10 mg/kg; MedChemExpress) or vehicle (10% dimethyl sulfoxide, 0.9% NaCl) during the last 3 weeks of diabetes (Fig. S4*A*). At 16 weeks of diabetes, mice were euthanized, and whole eyes or retina were extracted. All procedures were approved by the Penn State College of Medicine Institutional Animal Care and Use Committee and were in accordance with the Association for Research in Vision and Ophthalmology statement on the ethical use of animals in ophthalmological research.

### Cell culture

Human MIO-M1 Müller cells were obtained from the UCL Institute of Ophthalmology. MIO-M1 cells deficient for REDD1 (REDD1 KO) were generated by CRISPR–Cas9 genome editing as previously described (65). MIO-M1 cultures were maintained in Dulbecco's modified Eagle's medium (Thermo Fisher Scientific) containing 5.6 mM glucose and supplemented with 10% heat-inactivated fetal bovine serum and 1% penicillin–streptomycin. MIO-M1 cells stably expressing an shRNA targeting GSK3β were generated as previously described (27). Cells expressing pLKO.1-TRC (provided by David Root [Addgene Plasmid #10879]) were used as an shRNA control. To model hyperglycemia, culture medium was supplemented with d-glucose to achieve a final concentration of 30 mmol/l glucose. Alternatively, media were supplemented with 24.4 mM mannitol as an osmotic control. Cells were transfected using Lipofectamine 2000 (Life Technologies). Plasmids included pCMV5 vector, pCMV-HA-caGSK3β, and pRL-Renilla luciferase (Promega). The NF-κB-TATA luciferase reporter plasmid was kindly provided by Dr Edward Harhaj (Penn State College of Medicine). Where indicated, cell culture medium was supplemented with 1 μM VP3.15 (MedKoo Biosciences) or 1 μM CHIR99021 (Tocris).

### Immunofluorescence microscopy

Whole eyes were excised, and corneas were punctured, followed by incubation in 4% paraformaldehyde (PFA, pH 7.5) for 30 min. Eyes were washed with PBS and incubated at 4 °C in 30% sucrose solution containing 0.05% sodium azide. Eyes were embedded in optimal cutting temperature compound, flash frozen, and sectioned. Cryosections (10 μm) were fixed in 2% PFA, permeabilized in PBS with 0.1% Triton-X-100. To visualize NF-κB nuclear localization, MIO-M1 cells were cultured on chamber slides (CELLTREAT) for 24 h prior to exposure to hyperglycemia conditions. Cells were then fixed in 4% PFA and permeabilized with PBS with 0.1% Triton-X-100. Sections or cell monolayers were then blocked with 10% normal donkey serum and labeled with the appropriate antibodies (Table S1). Slides were mounted with Vectashield Plus Antifade Mounting Medium with DAPI mounting media (Vector labs). Images were captured using an SP8 confocal laser microscope (Leica Microsystems) with frame-stack sequential scanning. ImageJ (National Institutes of Health) was used to estimate macrophage counts from three separates fields of view per retina. Thresholds were set to isolate CD80- or F4/80-positive cells, and particles were counted. Single-stained population counts were also confirmed manually.

### Western blotting

Retinas were flash frozen in liquid nitrogen and homogenized as previously described (66). Retinal proteins were quantified by DC Protein Assay (Bio-Rad Laboratories). Equal amounts of protein from cell lysates or retinal homogenates were combined with Laemmli buffer, boiled for 5 min, and fractionated in Criterion Precast 4 to 20% gels (Bio-Rad Laboratories). Proteins were transferred to a polyvinylidene fluorine membrane, blocked with 5% milk in Tris-buffered saline Tween-20, and incubated overnight with the appropriate antibodies (Table S1). Antibody binding was visualized with enhanced chemiluminescence Clarity Reagent (Bio-Rad Laboratories) using a ProteinSimple Fluorochem E, and bands were quantified by densitometry using ImageJ.

### Luciferase reporter assay

Cells were cotransfected with NF-κB-TATA luciferase and pRL-Renilla luciferase plasmids as described previously. Transfection media were removed after 24 h, and cells were exposed to culture medium as indicated. Luciferase activity was measured on a FlexStation3 (Molecular Devices) using a Dual-Luciferase Assay Kit (Promega).

### PCR analysis

Total RNA was extracted with TRIzol (Invitrogen) according to the manufacturer’s protocol. RNA (1 μg) was reversed transcribed using the High-Capacity cDNA Reverse Transcription Kit (Applied Biosystems) and subjected to quantitative real-time PCR (QuantStudio 12K Flex Real-Time PCR System; Thermo Fisher Scientific; Research Resource Identifier; SCR_021098) using Quantitect SYBR Green Master Mix (Qiagen). PCR primer sequences are listed in Table S2. Changes in mRNA expression were normalized to GAPDH mRNA expression using the 2^−ΔΔCT^ method.

### DNA-binding ELISA

NF-κB activity was quantified using a colorimetric NF-κB p65 DNA-binding ELISA (Trans AM NF-κB p65; Active Motif) as described previously (27). Briefly, 40 μg of whole retinal homogenates was incubated for 2 h in the presence of an immobilized oligonucleotide encoding the NF-κB consensus sequence. Binding of the p65 subunit was quantified using an anti-p65 antibody and a horseradish peroxidase–conjugated secondary. The absorbance (λmax = 450 nm) was recorded using Spectra Max M5 plate reader (Molecular Devices).

### Statistical analysis

Data are expressed as mean ± SD. Data were analyzed by two-way ANOVA, and pairwise comparisons were made using the Tukey’s test for multiple comparisons. The relationships between REDD1 expression and blood glucose levels were tested by Pearson’s correlation analysis. Significance is indicated at *p* < 0.05 for all analyses. Exact *p* values for experimental groups with significantly different means are listed in Table S3.

## Data availability

All data for this publication are included in the article or are available from the corresponding author upon request.

## Supporting information

This article contains supporting information.

## Conflict of interest

The authors declare that they have no conflicts of interest with the contents of this article.
