# Supplementary material for: REDD1-dependent GSK3β dephosphorylation promotes NF-κB activation and macrophage infiltration in the retina of diabetic mice
Source: J Biol Chem. 2023 Jun 29;299(8):104991. doi: 10.1016/j.jbc.2023.104991 (PMC10407432; doi:10.1016/j.jbc.2023.104991)
Supplement: Supporting Information [file mmc1.pdf]

Supporting Information

REDD1-dependent GSK3β dephosphorylation promotes NF-κB activation and macrophage infiltration in the retina of diabetic mice

Sunilkumar et al.

Table of Contents:

1. Table S1: Antibody information

2. Table S2: PCR primer sequences

3. Table S3: Significant differences detected between experimental groups.

4. Figure S1: GSK3β knockdown in Müller glia cultures

5. Figure S2: Enhanced CD80 expression in the retina of diabetic mice required REDD1.

6. Figure S3. GSK3β inhibition in Müller glia cultures.

7. Figure S4: Diabetes-induced CD80 expression in the retina was attenuated by GSK3 inhibition.

Table S1. Antibody information

Antibody list					
Assay	Antibody	Source	Dilution	Catalog #	Lot#
Western blotting	REDD1	ProteinTech	1:500	10638-1-AP	95508
	phospho GSK3β (Ser 9)	Cell Signaling	1:1000	5558	10
	GSK3 α/β		1:1000	5676	4
	GSK3β		1:1000	12456	10
	phospho GS (Ser 641)		1:1000	3891	2
	phospho NF-κB (Ser 536)		1:1000	3033	17
	NF-κB		1:1000	8242	8
	IκBα		1:1000	4814	17
	phospho IKK α/β (Ser 176/180)		1:1000	2697	21
	IKKβ		1:1000	2678	2
	phospho IKKγ (Ser 376)		1:1000	2689	3
	p44/42 MAPK (Erk1/2) (Thr202/Tyr204)		1:1000	9101	29
	NF-κB2 p100/52		1:1000	4882	7
	CCL2/MCP-1		1:1000	2029	2
	HA-tag		1:1000	3724	10
	Actin		1:1000	4970	19
	GAPDH	Santa Cruz	1:2000	sc-47724	H1021
	Goat anti-Rabbit IgG h+l-HRP	Bethyl laboratories	1:10000	A120-101	44
	Goat anti-mouse IgG h+l-HRP		1:10000	A90-116	43
IF / IHC	B7-1/CD80	R&D systems	1:50	AF740	cvm0522031
	F4/80	Cell Signaling	1:50	30325	3
	Goat anti-Rabbit Alexafluor 647	Jackson	1:1000	711605152	159933
	Donkey anti-goat Alexafluor 488		1:1000	705546147	122088

Table S2. PCR primer sequences

PCR primer list			
Species	Target	Forward sequence (5' - 3')	Reverse sequence (5' - 3')
Mouse	REDD1	GGGATCGTTTCTCGTCCTCC	ATGAGGAGTCTTCCTCCGGC
	CCL2	CACTCACCTGCTGCTACTCA	GCTTGGTGACAAAAACTACAGC
	CCL5	TGCTGCTTTGCCTACCTCTC	TCCTTCGAGTGACAAACACGA
	GAPDH	GGTGGTCTCCTCTGACTTCAACA	GTTGCTGTAGCCAAATTCGTTGT
Human	CCL2	CATGAAAGTCTCTGCCGCCC	GGGCATTGATTGCATCTGGCTG
	CCL5	TGCTGCTTTGCCTACATTGC	CATCCTTGACCTGTGGACGA
	GAPDH	GTTGTCTCCTGCGACTTCA	TGCTGTAGCCGTATTCATTG

Table S3: Significant differences detected between experimental groups.

	Groups	Adjusted P Value
Figure 1E	REDD1+/+ Veh vs. REDD1+/+ STZ	0.0012
Figure 1E	REDD1+/+ STZ vs. REDD1-/- STZ	0.0149
Figure 1E	REDD1+/+ Veh vs. REDD1+/+ STZ	0.0009
Figure 1E	REDD1+/+ STZ vs. REDD1-/- STZ	0.0012
Figure 1F	REDD1+/+ Veh vs. REDD1+/+ STZ	0.0213
Figure 1F	REDD1+/+ STZ vs. REDD1-/- STZ	0.0393
Figure 2A	WT LG vs. WT HG	0.0333
Figure 2A	WT HG vs. REDD1 KO HG	0.0069
Figure 2B	WT LG vs. WT HG	0.0006
Figure 2B	WT HG vs. REDD1 KO HG	0.0398
Figure 2C	WT LG vs. WT HG	0.0036
Figure 2C	WT HG vs. REDD1 KO HG	0.0384
Figure 2E	WT LG vs. WT HG	<0.0001
Figure 2E	WT HG vs. REDD1 KO HG	0.0002
Figure 2F	EV LG vs. caGSK3 LG	0.0005
Figure 2F	EV HG vs. caGSK3 HG	0.0003
Figure 3A	shScr LG vs. shScr HG	<0.0001
Figure 3A	shScr HG vs. shGSK3 HG	<0.0001
Figure 3B	shScr LG vs. shScr HG	<0.0001
Figure 3B	shScr HG vs. shGSK3 HG	<0.0001
Figure 3D	shScr LG vs. shScr HG	<0.0001
Figure 3D	shScr HG vs. shGSK3 HG	<0.0001
Figure 3E	shScr LG vs. shScr HG	<0.0001
Figure 3E	shScr HG vs. shGSK3 HG	<0.0001
Figure 3F	shScr LG vs. shScr HG	<0.0001
Figure 3F	shScr HG vs. shGSK3 HG	<0.0001

	Groups	Adjusted P Value
Figure 4A	LG veh vs. HG veh	<0.0001
Figure 4A	HG veh vs. HG VP3.15	<0.0001
Figure 4B	LG veh vs. HG veh	<0.0001
Figure 4B	HG veh vs. HG VP3.15	<0.0001
Figure 4C	LG veh vs. HG veh	<0.0001
Figure 4C	HG veh vs. HG VP3.15	<0.0001
Figure 4D	LG veh vs. HG veh	0.0133
Figure 4D	HG veh vs. HG VP3.15	0.0004
Figure 4E	LG veh vs. HG veh	0.0019
Figure 4E	HG veh vs. HG VP3.15	0.0002
Figure 5A	shScr LG vs. shScr HG	0.0018
Figure 5A	shScr HG vs. shGSK3 HG	<0.0001
Figure 5B	shScr LG vs. shScr HG	<0.0001
Figure 5B	shScr HG vs. shGSK3 HG	<0.0001
Figure 5C	shScr LG vs. shScr HG	<0.0001
Figure 5C	shScr HG vs. shGSK3 HG	<0.0001
Figure 5D	LG veh vs. HG veh	0.0435
Figure 5D	HG veh vs. HG VP3.15	0.0385
Figure 5E	LG veh vs. HG veh	0.0045
Figure 5E	HG veh vs. HG VP3.15	0.0337
Figure 5F	LG veh vs. HG veh	<0.0001
Figure 5F	HG veh vs. HG VP3.15	<0.0001
Figure 6A	Veh DMSO vs. STZ DMSO	0.0036
Figure 6A	Veh VP3.15 vs. STZ VP3.15	0.0061
Figure 6B	Veh DMSO vs. STZ DMSO	0.011
Figure 6B	STZ DMSO vs. STZ VP3.15	0.0181
Figure 6C	Veh DMSO vs. STZ DMSO	0.0001
Figure 6C	STZ DMSO vs. STZ VP3.15	0.0054
Figure 6D	Veh DMSO vs. STZ DMSO	<0.0001
Figure 6D	STZ DMSO vs. STZ VP3.15	<0.0001
Figure 6E	Veh DMSO vs. STZ DMSO	0.0016
Figure 6E	STZ DMSO vs. STZ VP3.15	0.0053
Figure 6F	Veh DMSO vs. STZ DMSO	0.0058
Figure 6F	STZ DMSO vs. STZ VP3.15	0.0047
Figure 6H	Veh DMSO vs. STZ DMSO	<0.0001
Figure 6H	STZ DMSO vs. STZ VP3.15	<0.0001
Figure 6I	Veh DMSO vs. STZ DMSO	<0.0001
Figure 6I	STZ DMSO vs. STZ VP3.15	<0.0001

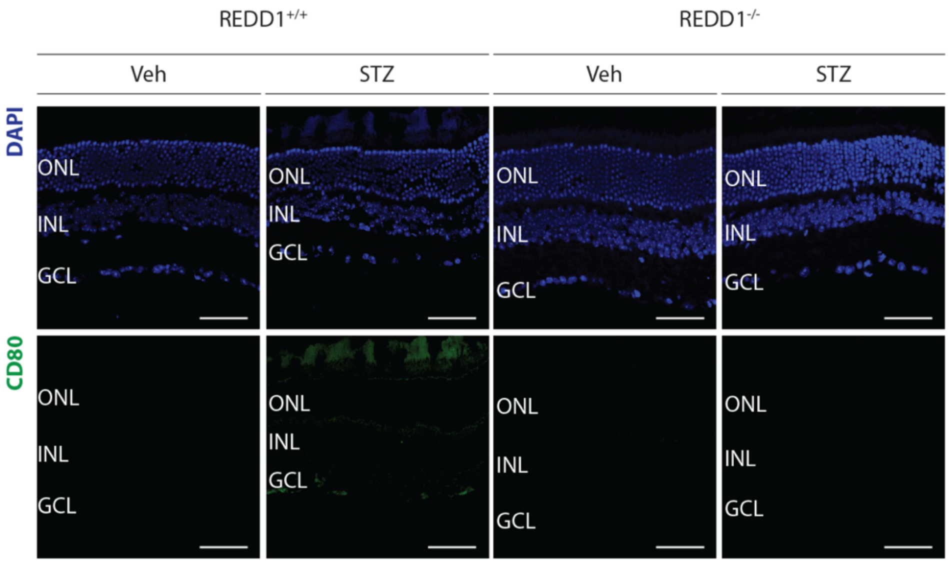

Figure S1. Enhanced CD80 expression in the retina of diabetic mice required REDD1. Diabetes was induced in REDD1$^{+/+}$ and REDD1$^{-/-}$ mice by administration of streptozotocin (STZ). All analyses were performed 16 weeks after mice were administered STZ or a vehicle (Veh). Retinas were stained for CD80 (*green*) and counterstained with DAPI (*blue*). Representative micrographs are shown (400X magnification; scale bar 50 µm). Composite images are also provided in Fig. 1B. ONL, outer nuclear layer; INL, inner nuclear layer; GCL, ganglion cell layer.

Figure S2

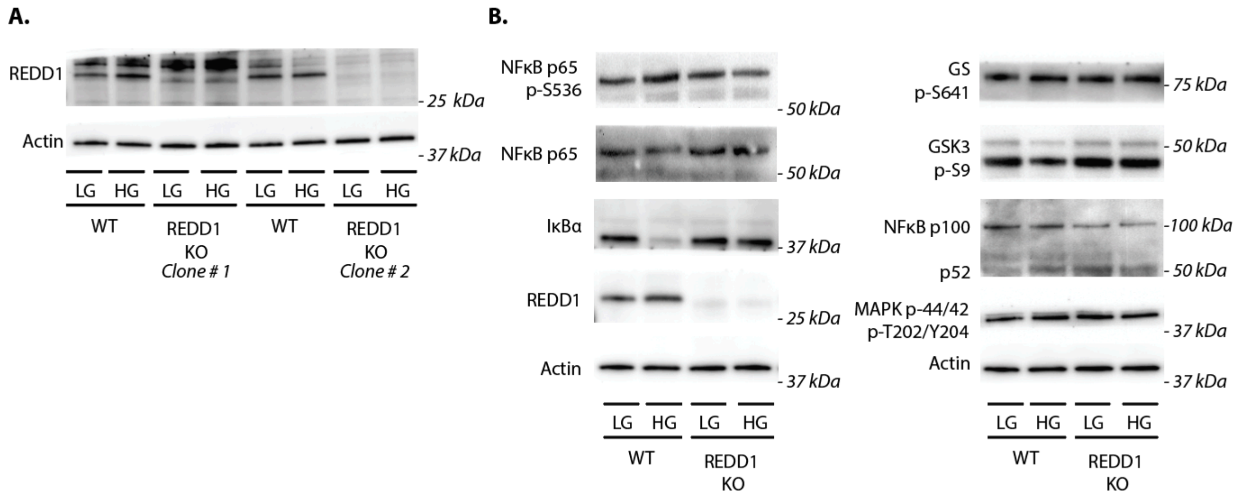

Figure S2. REDD1 is required for glucose induced NF-κB activation. Wild type (WT) and CRISPR REDD1 knock out (KO) MIO-M1 cells were exposed to medium containing 30 mM glucose (HG) or a low glucose osmotic control (LG; 5 mM glucose + 25 mM mannitol) for 24 h. *A*, REDD1 deletion in two separate clonal lines was validated by western blotting. All experiments in Figures 2-5 were performed in Clone #1. *B*, Canonical and non-canonical NF-κB signaling were analyzed in Clone #2 by western blotting. Representative blots are shown. Molecular mass (kDa) is indicated to the right of each blots.

Figure S3

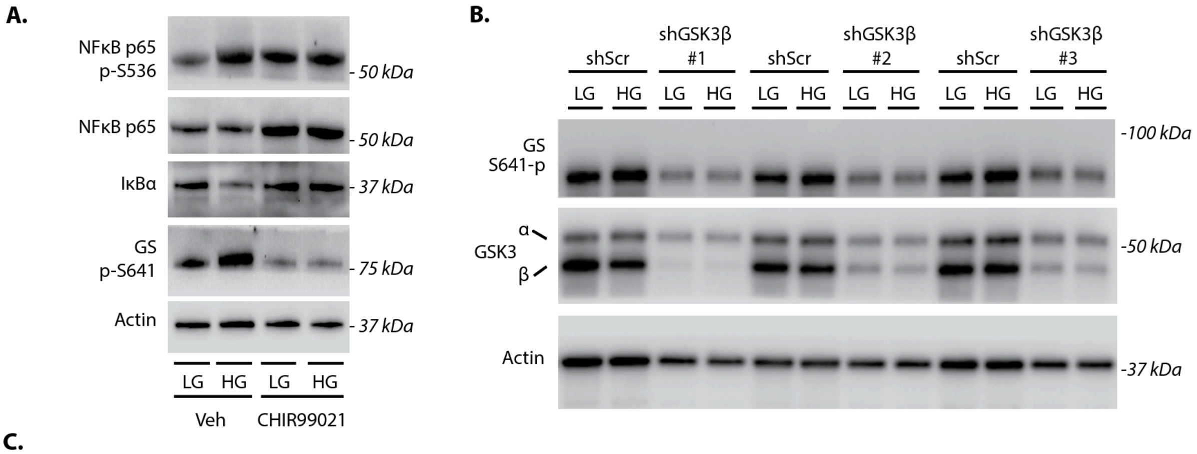

C.

shRNA	REFSEQ ID	Oligonucleotide sequence
shScr	pLKO.1-TRC control (RRID:Addgene_10879)	CCGCAGGTATGCACGCGT
shGSK3β #1	NM_002093.3,NM_001146156.1	CCGGCCCAAATGTCAAACTACCAAACTCGAGTTTGGTAGTTTGACATTTGGGTTTTT
shGSK3β #2	NM_002093.3,NM_001146156.1	CCGGCCGATTGCGTTATTTCTTCTACTCGAGTAGAAGAAATAACGCAATCGGTTTTT
shGSK3β #3	NM_002093.3,NM_001146156.1	CCGGCCAATGTTTCGTATATCTGTTCTCGAGAACAGATATACGAAACATTGGTTTTT

Figure S3. GSK3β inhibition in Müller glia cultures. *A*, NF-κB signaling was evaluated in cells in the presence or absence of a GSK3 selective inhibitor CHIR-99021 (1 μM). Cells were exposed to medium containing 30 mM glucose (HG) or a low glucose osmotic control (LG; 5 mM glucose + 25 mM Mannitol) for 24 h. Molecular mass are indicated to the *right* of the blots. *B*, western blotting was used to evaluate expression of GSK3 and phosphorylation of glycogen synthase (GS) in human MIO-M1 cell lines stably expressing shRNAs targeting GSK3β (shGSK3β) or a control shRNA (shScr). *C,* oligonucleotide sequences and Reference Sequence Identifiers (REFSEQ ID) for shRNAs.

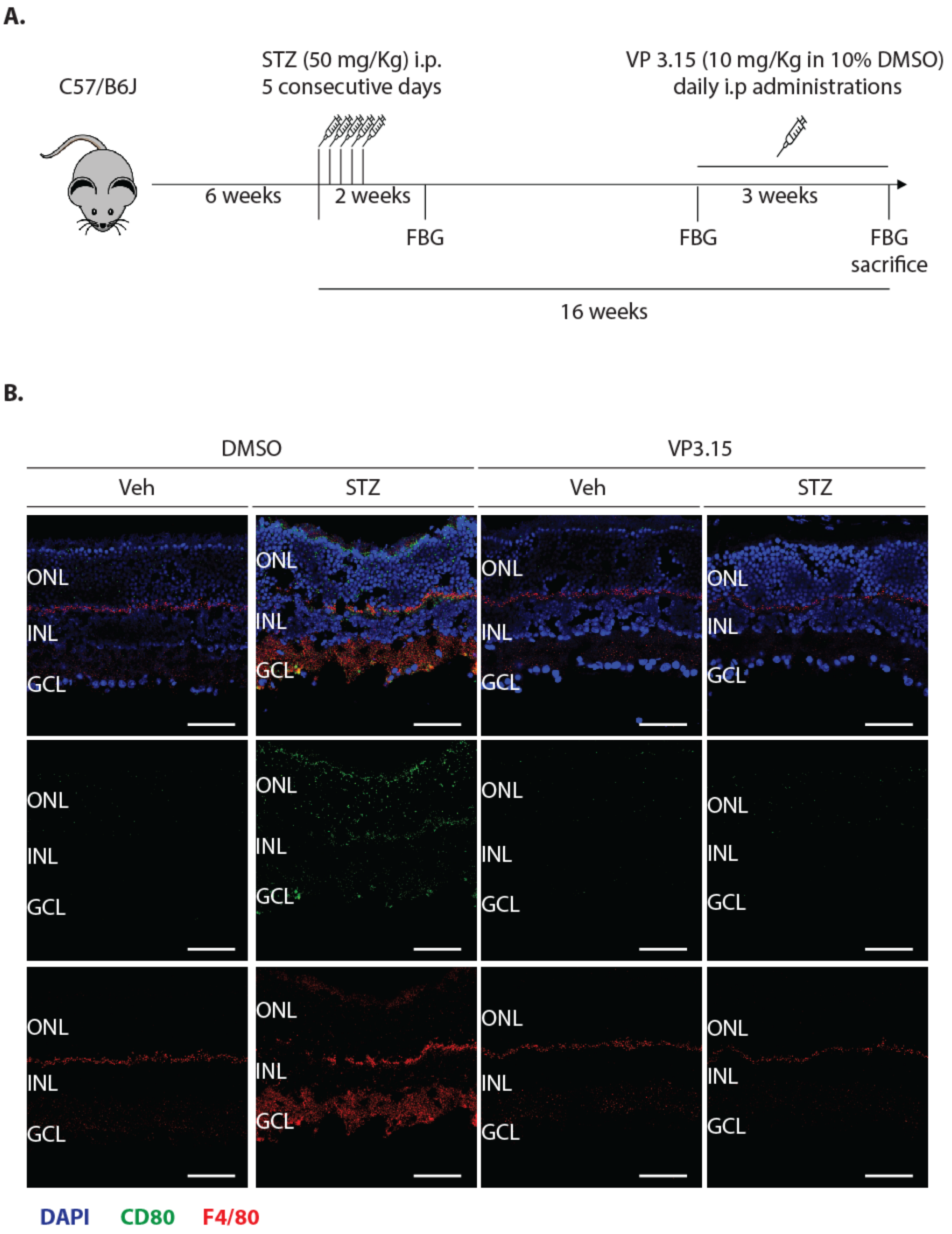

Figure S4. Diabetes-induced CD80 expression in the retina was attenuated by GSK3 inhibition. *A*, Model illustrating experiment paradigm wherein diabetes was induced in C57/B6J mice by administration of streptozotocin (STZ). All analyses were performed 16 weeks after mice were administered STZ or a vehicle (Veh). During the 3 weeks prior to sacrifice, mice were administered daily intraperitoneal injections of either VP3.15 (10mg/Kg in 10% DMSO) or a vehicle (10% DMSO). *B*, Retinal cryosections were stained for CD80 (*green*), F4/80 (*red*) and nuclei were counterstained with DAPI (*blue*). Representative micrographs with individual channels are shown (400X magnification; scale bar 50 µm). Composite images are also provided in Fig. 6H with quantification of CD80 and F4/80 in Figs. 6I and 6J, respectively. ONL, outer nuclear layer; INL, inner nuclear layer; GCL, ganglion cell layer.
